# Evaluation of image quality in mobile cone‐beam CT with dose modulation using automatic exposure control: A phantom study

**DOI:** 10.1002/acm2.70248

**Published:** 2025-09-28

**Authors:** Keita Okazaki, Wenchao Cao, Reza Taleei, Firas Mourtada, Jun Li, Karen Mooney, Pramila Rani Anne, Yingxuan Chen

**Affiliations:** ^1^ Department of Radiation Oncology Thomas Jefferson University Philadelphia Pennsylvania USA

**Keywords:** automatic exposure control, CBCT, HDR brachytherapy, image quality

## Abstract

**Background:**

The integration of mobile cone‐beam computed tomography (CBCT) into brachytherapy workflows offers clinical advantages such as immediate verification of applicator placement and adaptive treatment planning. These benefits require sufficient image quality to delineate applicators, target volumes, and organs at risk. A systematic evaluation of automatic exposure control (AEC) settings, radiation dose, and image quality is essential to ensure clinically acceptable imaging while minimizing patient exposure.

**Purpose:**

This study evaluates the characteristics of AEC and its impact on image quality and radiation dose in a mobile CBCT system used for brachytherapy.

**Methods:**

The Elekta ImagingRing CBCT system was used to scan a CatPhan phantom under two imaging protocols: Medium Dose Limit (MDL) and Ultra‐High Dose Limit (UHDL). This system employs a two‐layer mAs modulation process, consisting of preset mA values based on body mass index (BMI) and adjusted mA based on real‐time AEC. A bolus was used to simulate larger patient sizes. Real‐time x‐ray tube current at 10 degrees intervals was recorded. Image quality was evaluated using image noise, noise power spectrum (NPS), modulation transfer function (MTF), Hounsfield Unit (HU) linearity, uniformity index (UI), and contrast‐to‐noise ratio (CNR) across different protocols.

**Results:**

AEC effectively modulated x‐ray tube current in the MDL protocol after x‐ray attenuation through the scanned phantom was measured. The UHDL protocol demonstrated greater noise reduction than the MDL. MTF values were comparable between protocols, indicating preserved spatial resolution in the MDL protocol. HU linearity was consistent across all protocols, with *R* ^2^ > 0.993.

**Conclusion:**

AEC in mobile CBCT optimized radiation dose and image quality by adjusting tube current based on attenuation. The MDL protocol reduced radiation exposure while maintaining image quality, making it a viable option for verifying applicator placement and treatment planning in brachytherapy. The UHDL protocol achieved noise reduction with the maximum available tube current.

## 1 INTRODUCTION

Brachytherapy is a crucial treatment modality in modern radiation oncology, particularly for gynecologic, prostate, and breast cancer.^1^, ^2^, ^3^ This technique involves placing radioactive sources directly within or near the target volume, allowing for highly localized dose delivery while minimizing radiation exposure to surrounding healthy tissues. Precise source placement is essential for achieving optimal dose distribution, making imaging a fundamental component of both treatment planning and real‐time procedural guidance.^4^, ^5^ Three‐dimensional (3D) imaging modalities, such as computed tomography (CT) and magnetic resonance imaging (MRI), have significantly enhanced visualization and target definition. The introduction of 3D imaging has markedly improved the accuracy of brachytherapy planning and delivery.^6^

Cone‐beam computed tomography (CBCT) was initially developed for image guidance in external beam radiotherapy (EBRT) and has since been adapted for intraoperative 3D imaging in brachytherapy.^7^ Mobile CBCT systems are increasingly being integrated into operating rooms to provide real‐time imaging during brachytherapy applicator placement, eliminating the need to transfer patients to a separate CT scanner. This integration streamlines workflow and enhances procedural efficiency.^8^

To optimize imaging quality and imaging dose in CBCT, automatic exposure control (AEC) has been incorporated into these systems. AEC dynamically adjusts the x‐ray tube current based on patient anatomy, a feature that has been a standard in diagnostic CT imaging, where its benefits in dose optimization are well documented.^9^ However, the role of AEC in mobile CBCT systems for brachytherapy remains less well‐studied. The application of AEC in this setting has the potential to enhance image quality by balancing radiation dose and image noise in real time.^10^

The integration of mobile CBCT into brachytherapy workflows provides several clinical advantages. First, it enables immediate verification of applicator placement without requiring patient transfer to a conventional CT scanner, thereby reducing procedural time and improving workflow efficiency. Second, real‐time imaging facilitates adaptive treatment planning, allowing for corrections in applicator positioning or adjustments to account for anatomical changes before the radioactive source is loaded. This capability is particularly beneficial in high‐dose‐rate (HDR) brachytherapy, where precise dose conformity is critical.^11^, ^12^ However, achieving these benefits necessitates that mobile CBCT systems provide sufficient image quality to accurately delineate applicators, target volumes, and organs at risk (OARs). Therefore, a systematic evaluation of the relationship between AEC settings, radiation dose, and image quality in mobile CBCT systems is essential to ensure clinically acceptable imaging while minimizing patient exposure.^13^

Previous studies evaluated the image quality of this mobile CBCT system using different scan protocols which had different exposure time, voltage, and tube output. They obtained good image quality with a Catphan phantom using different protocols.^14^ However, to our knowledge, there has not yet been any study evaluating image quality of a mobile CBCT with x‐ray tube current modulation using AEC.

In this study, we aim to assess the performance of AEC in a mobile CBCT system by simulating various patient body mass index (BMI) conditions. Moreover, we comprehensively evaluate image quality using a phantom with and without a bolus across different BMI settings under two imaging protocols.

## 2 MATERIALS AND METHODS

### 2.1 Image acquisition and phantom

The Elekta ImagingRing CBCT system (Elekta, Veenendaal, Netherlands) was installed at our institution for brachytherapy in 2022.^12^ The system was upgraded to version 2.11.6 in December 2024 to further improve image quality and was evaluated in this study. This system operates at 120 kVp and includes two imaging protocols: the Medium Dose Limit (MDL) protocol and the Ultra‐High Dose Limit (UHDL) protocol. Also, this system has two layers of mAs modulation: the preset mA based on BMI and real‐time automatic exposure control (AEC) adjustments. Pelvis vagina small field of view (SFOV) full scan, which we use at our institution for HDR patients, was performed in this study. The x‐ray tube and detector were rotated simultaneously to acquire the projections among 360 degrees for CBCT reconstruction. In this study, we created four simulation patients with BMI 20, 26, 34, and > 40 as shown in Table 1.

**TABLE 1:** Details about simulated patient data.

Simulated patient name	Height	Weight	BMI
BMI 20	5'4"	120 lbs	20
BMI 26	5'4"	150 lbs	26
BMI 34	5'4"	200 lbs	34
BMI 40	5'4"	240 lbs	>40

As shown in Table 2, in the MDL protocol, the preset tube current (mA) values are determined based on BMI, with four preset values: 8.7 mA for a BMI of 20, 15.8 mA for a BMI of 26, and 17.1 mA for BMIs of 34 and 40. In the UHDL protocol, the tube current is fixed at 17.1 mA, regardless of BMI. In the MDL protocol, AEC dynamically adjusts the real‐time x‐ray tube current after acquiring information on x‐ray attenuation through the scanned object. In contrast, the UHDL protocol maintains a fixed maximum tube current of 17.1 mA, regardless of x‐ray attenuation.

**TABLE 2:** Preset tube current based on BMI.

	Preset tube current (mA)	
BMI	The MDL protocol	The UHDL protocol
20	8.7	17.1
26	15.8	17.1
34	17.1	17.1
>40	17.1	17.1

**FIGURE 1:**
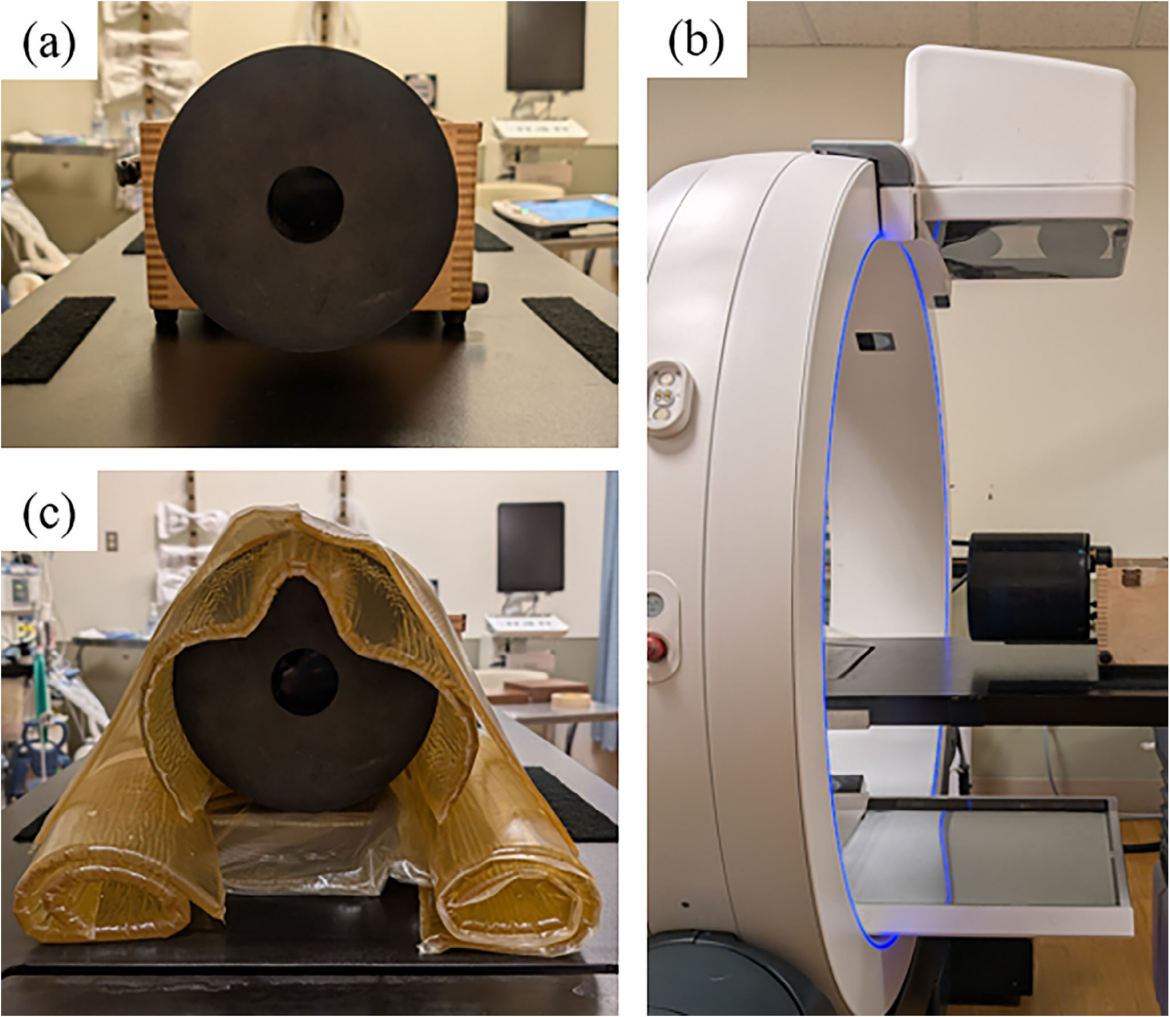
A CatPhan 500 phantom: (a) and (b) show the front and side view of the phantom without a bolus. (c) The front view with a bolus.

To evaluate image quality, a CatPhan 500 phantom (Phantom Laboratory, Salem, New York, USA) was used, as shown in Figure 1a.^15^ This phantom consists of four modules: CTP404, CTP528, CTP515, and CTP486. The CTP404 module is utilized for measuring slice width, sensitometry, and pixel size. The CTP528 module contains a 21‐line‐pair‐per‐centimeter gauge and a point source for high‐contrast resolution analysis. The CTP515 module includes supra‐slice and subslice contrast targets for evaluating low‐contrast resolution, while the CTP486 module is used to assess image uniformity. To simulate large patient size, the phantom was wrapped with a bolus, as shown in Figure 1b. The CatPhan 500 phantom diameter is 20 cm. After adding the additional bolus, the estimated phantom dimensions are approximately 28 cm in height and 32 cm in width. For all scans, The CBCT images were reconstructed with an in‐plane pixel size of 0.6 mm × 0.6 mm and a slice thickness of 0.6 mm.

### 2.2 AEC characteristics

The Elekta ImagingRing CBCT system can record real‐time tube current values at every 10‐degree projection angle throughout the scan. To evaluate the AEC response, real‐time mA values were acquired for each BMI preset under both protocols, both with and without a bolus wrapped around the phantom. When no bolus was applied, real‐time mA values were recorded for all BMI presets in both protocols. When a bolus was applied, real‐time mA values were obtained for each BMI preset in the MDL protocol, as well as for BMI 20 and BMI 40 in the UHDL protocol.

The recorded Weighted Cone Beam Dose Index (CBDI_w_) values were extracted from the CBCT system to assess radiation dose variation under different scanning conditions.

### 2.3 Image quality

To comprehensively evaluate image quality, we performed analysis for image noise, spatial resolution, HU linearity, uniformity index (UI), and contrast‐to‐noise ratio (CNR) in this study.

#### 2.3.1 Image noise

**FIGURE 2:**
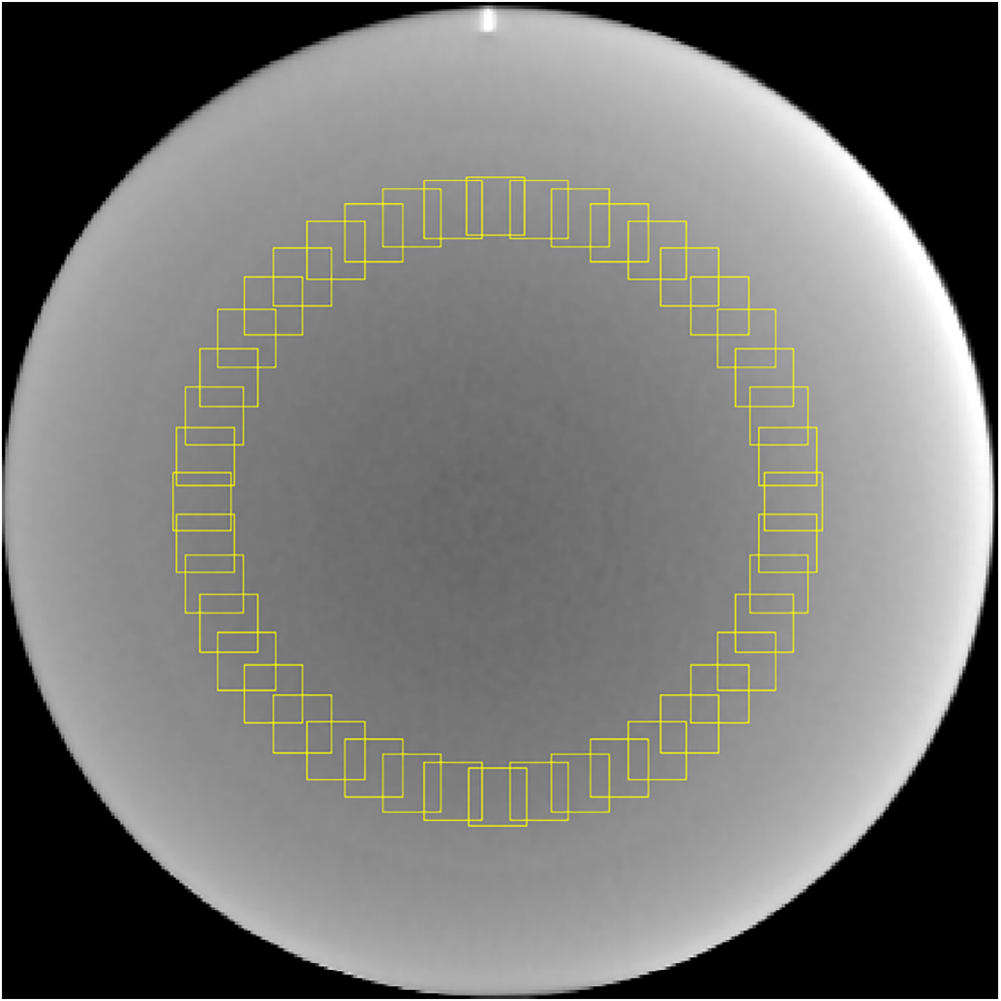
Forty‐four ROIs in the uniformity module. (WL/WW [‐40, 500]).

The CTP486 module was used to assess image noise and the noise power spectrum (NPS).^16^, ^17^ Forty‐four square regions of interest (ROIs), each measuring 20 × 20 pixels and corresponding to a physical area of 12 × 12 mm^2^, were placed radially at a 60 mm distance from the center of the phantom, as illustrated in Figure 2. Image noise was quantified as the mean standard deviation (SD) across all ROIs.

To analyze the NPS, the average HU value in each ROI was calculated, and the difference between individual pixel HU values and the ROI mean HU value was determined. A two‐dimensional Fourier Transform was then applied to these differences, and the NPS was computed using the following equation:

(1) $$\begin{array}{cc} & NPS\left(f_{x},f_{y}\right)=\frac{1}{N}\times \frac{D_{x}D_{y}}{V_{x}V_{y}}\sum_{i=1}^{N}\\ & \;\left|FFT_{2D}{\left\{ROI_{i}\left(x,y\right)-\bar{ROI_{i}\left(x,y\right)}\right\}}^{2}\right| \end{array}$$

where *N* represents the number of ROIs, *D_x_* and *D_y_* correspond to the pixel dimensions in the x and y directions, and *V_x_* and *V_y_* indicate the total pixel counts in the x and y directions.

#### 2.3.2 Spatial resolution

**FIGURE 3:**
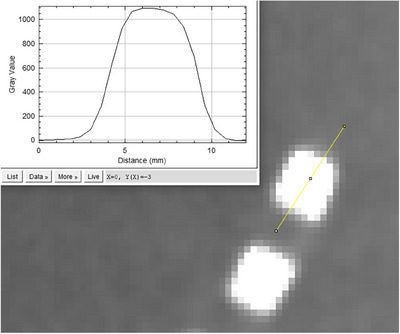
Analysis of MTF using the edge spread function. (WL/WW [40, 800]).

To evaluate spatial resolution, the modulation transfer function (MTF) was analyzed using the CTP528 module with an edge‐spread function (ESF) analysis.^18^, ^19^ A straight line was placed across the first line pair in the module, and distance and pixel intensity were measured using ImageJ, as illustrated in Figure 3.

The ESF was defined as the gray‐value transition within a range extending from eight pixels away from the first maximum intensity to the first maximum intensity. The line spread function (LSF) was then obtained by differentiating the ESF with respect to distance. Finally, a Fourier Transform was applied to the LSF to compute the MTF, providing a quantitative assessment of spatial resolution.

#### 2.3.3 HU linearity

**FIGURE 4:**
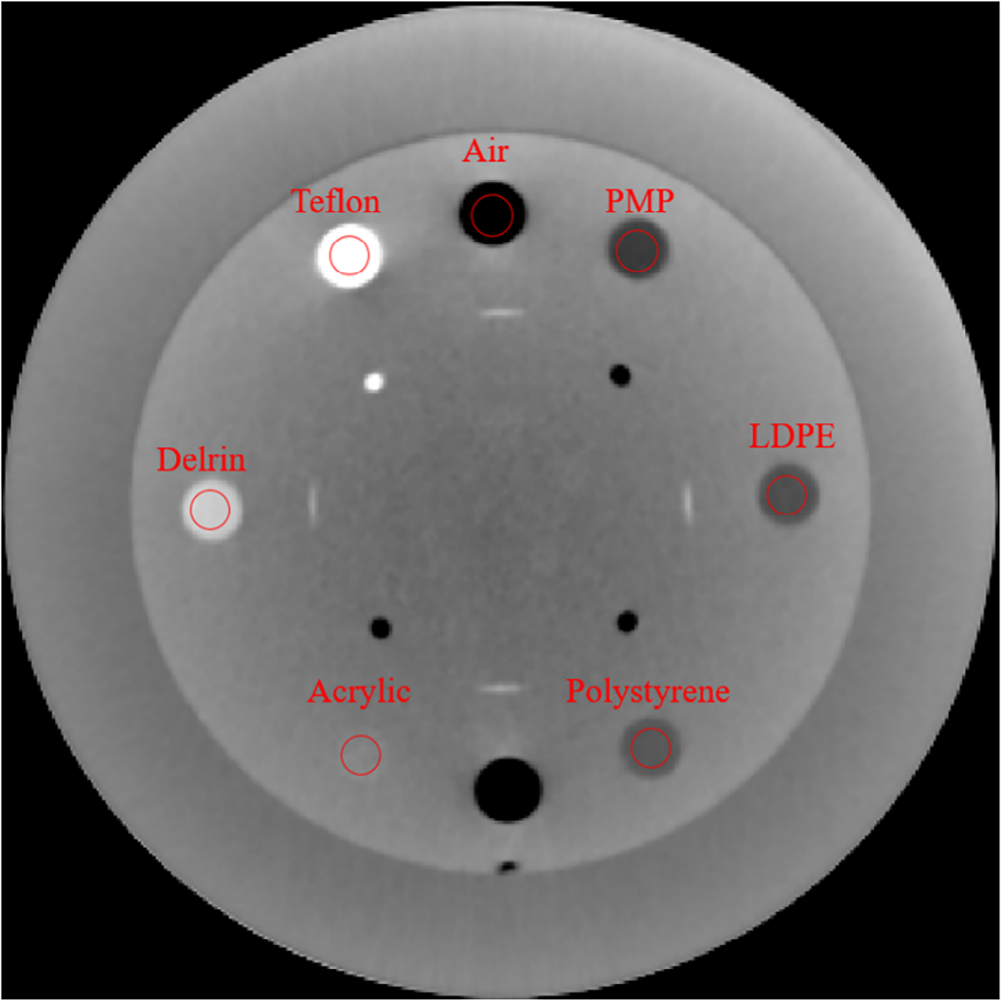
The ROI in different materials: air (‐1000 HU), PMP (‐200 HU), LDPE (‐100 HU), polystyrene (‐35 HU), acrylic (120 HU), Delrin (340 HU), and Teflon (990 HU). (WL/WW [0, 600]).

The CTP401 module was used to assess Hounsfield Unit (HU) linearity. This module contains seven materials with different electron densities, including air, polymethylpentene (PMP), low‐density polyethylene (LDPE), polystyrene, acrylic, Delrin, and Teflon. To evaluate HU values, circular ROIs with an 8 mm diameter were placed in each material, as illustrated in Figure 4. The mean HU values and SDs were measured using ImageJ, with a window level of 0 and a window width of 600.

HU linearity was assessed by calculating the coefficient of determination (*R* ^2^ value) using linear regression analysis, where the measured mean HU values were plotted against the expected HU values. The *R* ^2^ value was defined as follows:

(2) $$R^{2}=1-\frac{\sum_{i=1}^{n}{\left(y_{i}-\hat{y_{i}}\right)}^{2}}{\sum_{i=1}^{n}{\left(y_{i}-\hat{y}\right)}^{2}}$$

where $y_{i}$ represents the measured mean HU value, $\hat{y_{i}}$ denotes the predicted HU value from the trendline, and $\hat{y}$ corresponds to the average measured mean HU value.

#### 2.3.4 Uniformity

Uniformity was assessed using the CTP486 module by placing five square ROIs of 20 × 20 pixels. One ROI was positioned at the center of the image, while the remaining four were placed 60 mm from the center in the superior, inferior, right, and left directions to represent the peripheral ROIs. The UI was calculated using the following equation:

(3) $$UI=100\frac{{\bar{HU}}_{Periphery}-{\bar{HU}}_{Center}}{{\bar{HU}}_{Center}+1000}$$

where ${\bar{HU}}_{Center}$ and ${\bar{HU}}_{Periphery}$ represent the mean Hounsfield Unit in the central and peripheral ROIs, respectively. For ${\bar{HU}}_{Periphery}$, the peripheral ROI with the largest absolute difference from the central ROI was used.^20^, ^21^

#### 2.3.5 Contrast‐to‐noise ratio

To evaluate the CNR, three inserted materials—LDPE, polystyrene, and Delrin—in the CTP401 module were analyzed. The same ROIs defined in Section II.C.3 were used for these materials. Additional ROIs of identical size were placed in homogeneous background regions adjacent to the inserted materials. The CNR was calculated using the following equation:

(4) $$CNR_{Insert}=\frac{\left|{\bar{HU}}_{Insert}-{\bar{HU}}_{Background}\right|}{\sqrt{\frac{1}{2}\cdot \left(\sigma_{Background}^{2}+\sigma_{Insert}^{2}\right)}}$$

where $\bar{HU}$ denotes the mean Hounsfield Unit within the ROI, and σ represents the corresponding SD. This formulation accounts for the combined noise contributions from both the insert and background ROIs.^14^, ^20^

## 3 RESULTS

**FIGURE 5:**
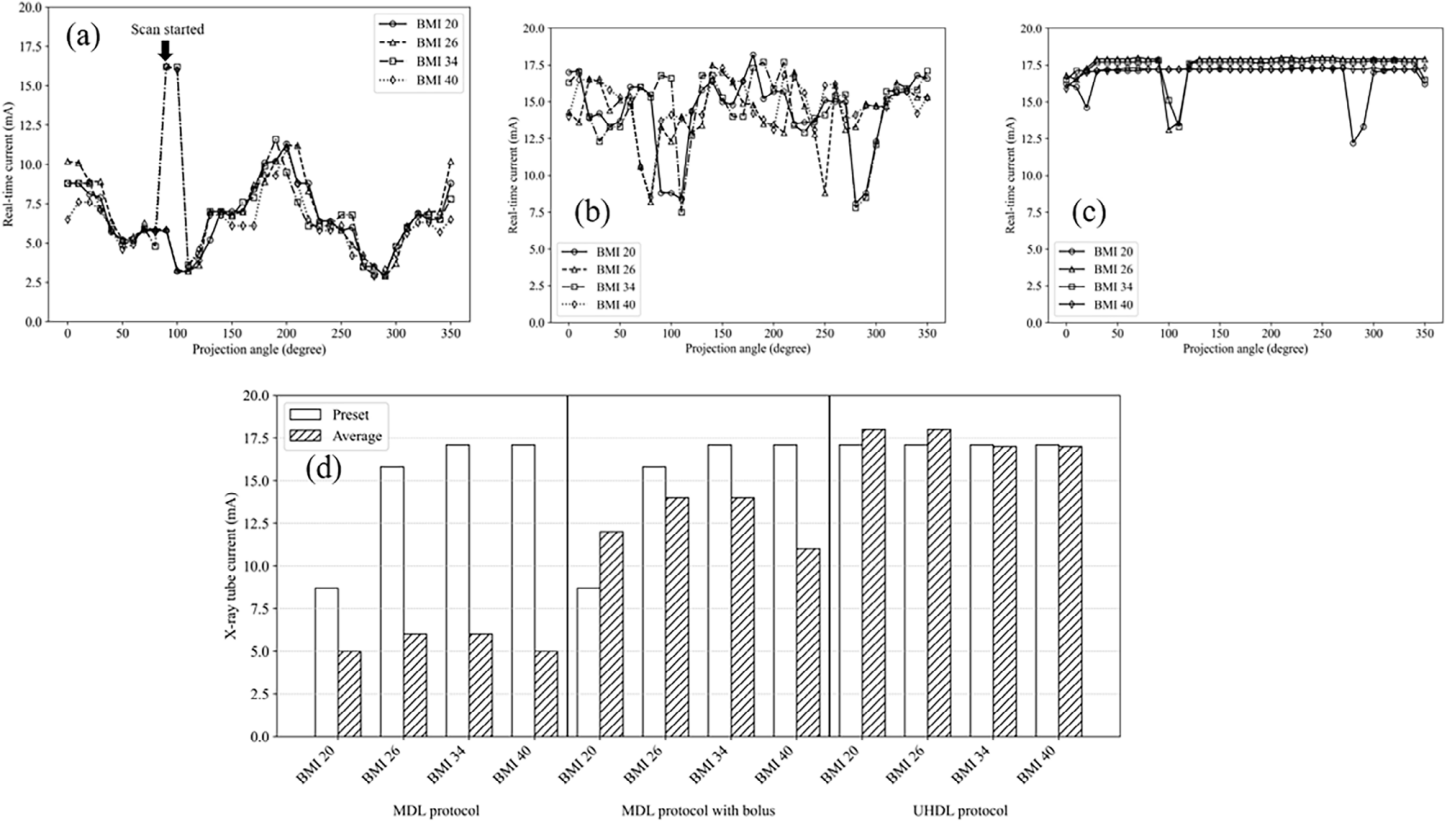
The real‐time tube current: (a) in the MDL protocol without a bolus, (b) in the MDL protocol with a bolus, (c) in the UHDL protocol without a bolus, and (d) shows preset mAs and average current.

To evaluate x‐ray tube current modulation during the image acquisition, real‐time x‐ray tube current at every 10‐degree projection angle were recorded and shown in Figure 5. Figure 5 illustrates how AEC modulates the tube current based on the preset mA values and the size of the scanned phantom.

Figure 5a presents the modulation trend in the MDL protocol using the CatPhan 500 phantom without a bolus. In this protocol, the preset mA values were 8.7 mA for a BMI of 20, 15.8 mA for a BMI of 26, and 17.1 mA for BMIs of 34 and 40. A dramatic increase at the start of the scan was observed for BMI34 and 40 in Figure 5a, as the first actual mA was determined by the presets of these BMI. After acquiring x‐ray attenuation data from the initial two scans, AEC adjusted the tube current accordingly.

To simulate a larger patient, the phantom was wrapped with a bolus to increase x‐ray attenuation. Figure 5b illustrates the real‐time current modulation when the phantom was wrapped in a bolus. AEC detected the increased attenuation due to the bolus and adjusted the tube current accordingly to compensate.

In contrast, the UHDL protocol maintains a fixed mA preset of 17.1 mA for all BMI categories, and the real‐time current in this protocol is shown in Figure 5c. The real‐time current decreased at a few points in Figure 5c since the current was affected by other factors such as anode heat, cooling system and so on. However, the average real‐time current remained constant as shown in Figure 5d.

Figure 5d shows the difference between the preset mA based on BMI and the average x‐ray tube current. Without a bolus, the MDL protocol decreased the current based on the scanned phantom. On the other hand, the MDL protocol increased the current when the phantom was wrapped by a bolus. There was no difference between the preset mA and the average current in the UHDL protocol.

The Weighted Cone Beam Dose Index (CBDI_w_) values recorded by the CBCT system, along with the average tube currents, are summarized in Table 3. The UHDL protocol exhibited consistently higher CBDI_w_ values due to its fixed maximum mA settings. In the MDL protocol, CBDI_w_ varied according to phantom size, with larger phantoms requiring higher radiation doses to maintain image quality.

**TABLE 3:** The recorded CBDI_w_ with and without a bolus for each BMI in two protocols.

CBDI_w_ (mGy)	BMI 20	BMI 40
MDL protocol	6.89	7.08
UHDL protocol	24.13	22.89
MDL protocol with bolus	15.79	15.34
UHDL protocol with bolus	22.69	22.66

**FIGURE 6:**
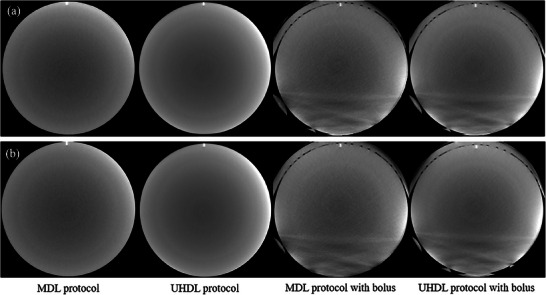
The images of the uniform module: (a) for BMI 20, and (b) for BMI40. (WL/WW [‐40, 500]).

Image noise was quantified by calculating the mean SD from 44 square ROIs, positioned radially at a 60 mm distance from the phantom center. The images of the uniformity module for BMI 20 and 40 in each protocol with and without a bolus are shown in Figure 6. The average SD values, presented in Table 4, indicate that the UHDL protocol resulted in lower image noise compared to the MDL protocol. In the MDL protocol, noise reduction was achieved due to AEC modulation of the real‐time tube current. When a bolus was applied, image noise was higher in the MDL protocol than in the UHDL protocol.

**TABLE 4:** Image noise analysis: The standard deviation with the average current.

	BMI 20		BMI 40	
	Average current (mA)	Image noise	Average current (mA)	Image noise
MDL protocol	5.0	7.446	5.0	7.190
UHDL protocol	18.0	7.090	17.0	7.192
MDL protocol with bolus	12.0	9.848	11.0	9.720
UHDL protocol with bolus	17.0	9.621	17.0	9.637

**FIGURE 7:**
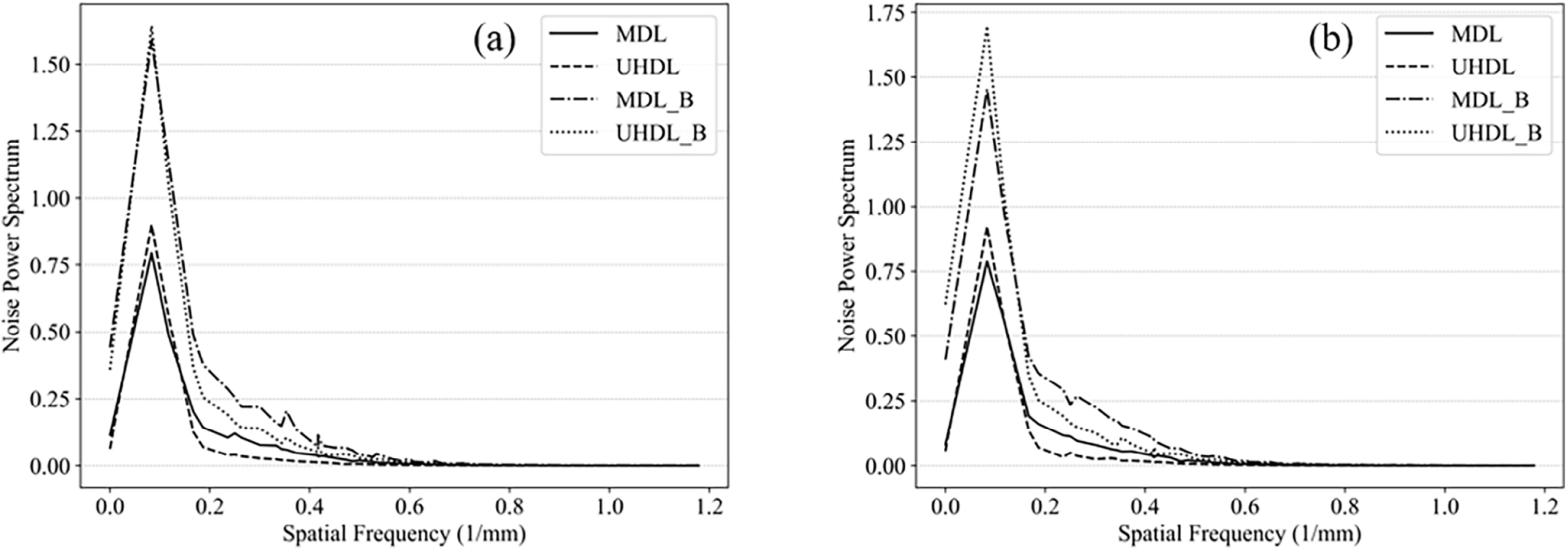
The noise power spectrum in two protocols showing the analyzed images of CTP486 module: (a) for BMI 20, and (b) for BMI40.

The NPS was analyzed for BMI 20 and BMI 40, as shown in Figure 7a and b. For BMI 20, the UHDL protocol without a bolus exhibited lower NPS values across all spatial frequencies. In contrast, the MDL protocol with a bolus demonstrated higher NPS values at all frequencies. The order of NPS values, from highest to lowest, correlated with the observed image noise levels. For BMI 40, the UHDL protocol exhibited higher NPS values at low spatial frequencies compared to the MDL protocol. This finding aligns with the image noise results, which showed that the MDL protocol had lower noise than the UHDL protocol in this BMI category.

**FIGURE 8:**
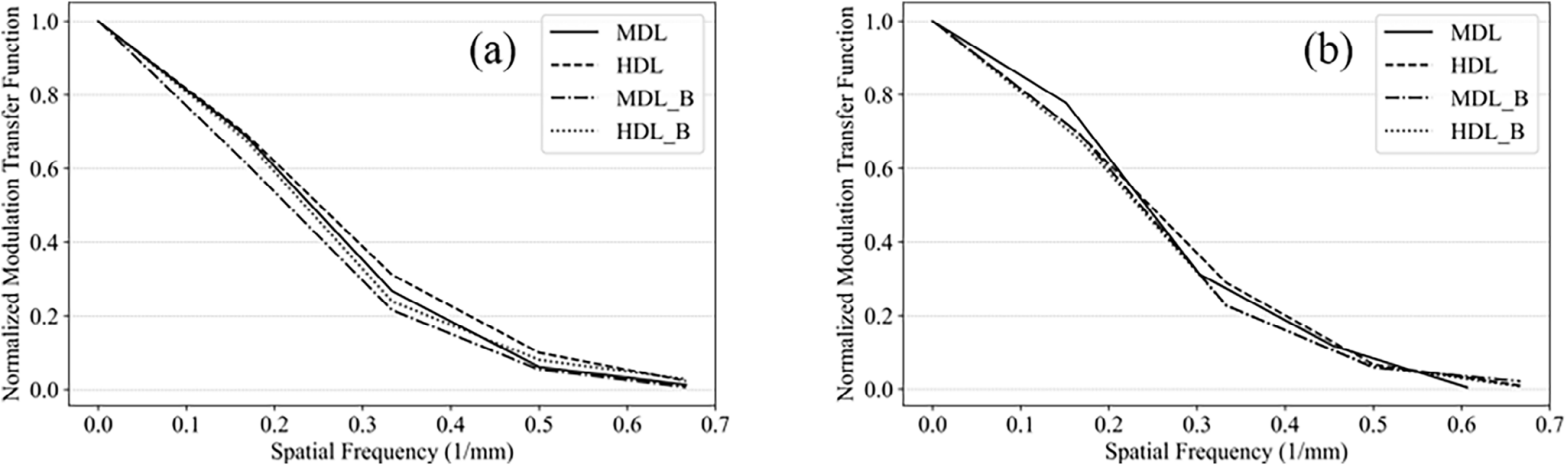
MTF curve with and without a bolus in two protocols: (a) for BMI20 and (b) for BMI 40.

The MTF was analyzed for BMI 20 and BMI 40, as shown in Figure 8a and b. MTF trends were comparable between the MDL and UHDL protocols. When a bolus was applied, MTF trends in both protocols remained similar at low spatial frequencies. However, at high spatial frequencies, the MTF for BMI 20 in the MDL protocol with a bolus was lower than in the UHDL protocol. Conversely, for BMI 40, the MTF in the MDL protocol with a bolus was higher at high frequencies than in the UHDL protocol.

**FIGURE 9:**
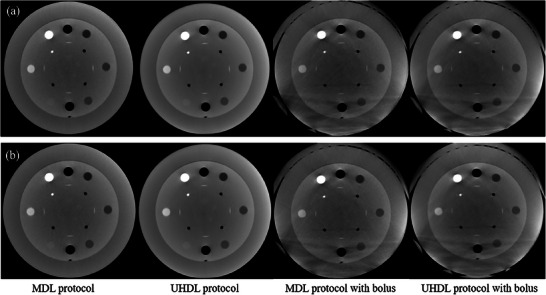
The images of the sensitometry module: (a) for BMI 20, and (b) for BMI40. (WL/WW [0, 600]).

**FIGURE 10:**
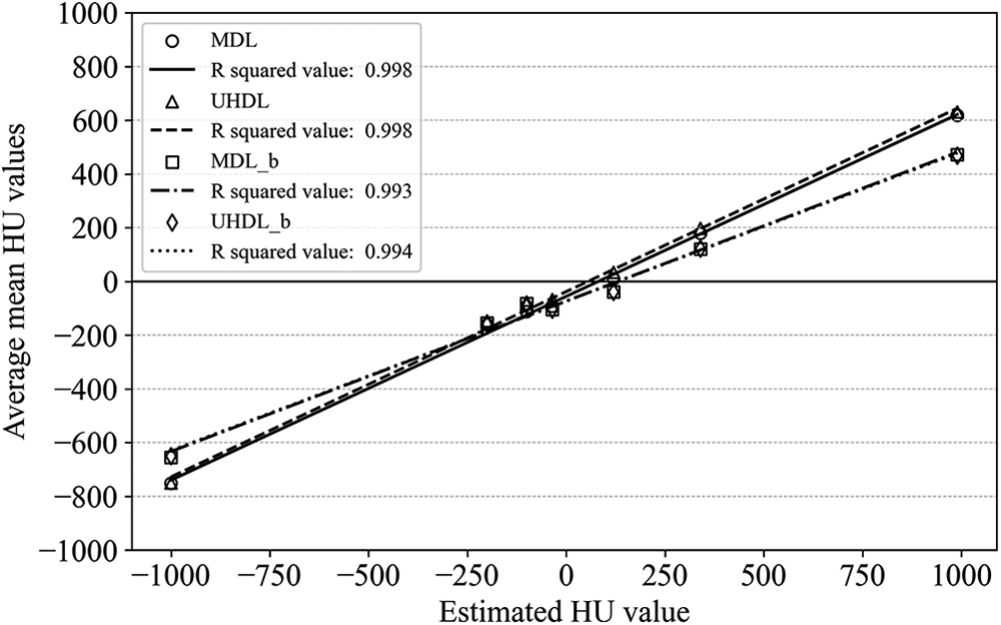
HU linearity for BMI 20 with and without a bolus in two protocols.

The assessment of image sensitometry was conducted using the CTP401 module as shown in Figure 9. The coefficient of determination (*R* ^2^ values) was calculated to evaluate the correlation between measured and expected mean HU values. Figure 10 shows the HU linearity for BMI 20 with and without a bolus in two protocols. As shown in Table 5, the *R* ^2^ values exceeded 0.993 across all BMI categories in both protocols, indicating strong HU linearity. When a bolus was applied, the *R* ^2^ values remained similar in both the UHDL and MDL protocols, confirming the robustness of HU linearity under different imaging conditions.

**TABLE 5:** The *R* ^2^ values for each BMI in two protocols.

	BMI 20	BMI 26	BMI34	BMI40
MDL protocol	0.998	0.996	0.999	0.998
UHDL protocol	0.998	0.997	0.996	0.998
MDL protocol with bolus	0.993	0.994	0.995	0.993
UHDL protocol with bolus	0.994			0.993

Table 6 summarizes the UI and CNR values obtained from the Catphan phantom images. Cupping artifacts were observed, and the artifacts commonly cited as a limitation of CBCT imaging.^14^ However, no significant differences in UI were found across all protocols. Regarding CNR, the UHDL protocol consistently demonstrated the highest values across all materials (LDPE, polystyrene, and Delrin), indicating improved low‐contrast detectability under higher‐dose conditions. In contrast, the use of a bolus led to a notable decrease in CNR values for all materials, regardless of BMI. This decline may be attributed to increased scatter and attenuation effects introduced by the bolus, which degrade image contrast and increase noise.

**TABLE 6:** The uniformity index (UI) and contrast‐to‐noise ratio (CNR) with BMI 20 and 40 in each protocol with and without bolus.

		CNR		
BMI‐Protocol	UI(%)	LDPE	Polystyrene	Delrin
BMI20				
‐MDL	8.32	22.07	17.87	24.55
‐UHDL	8.17	22.99	20.87	25.29
‐MDL with bolus	9.06	11.34	9.87	9.89
‐UHDL with bolus	8.85	13.78	10.48	10.57
BMI40				
‐MDL	7.19	20.88	18.15	23.42
‐UHDL	8.49	21.49	19.05	24.07
‐MDL with bolus	8.26	10.76	9.17	10.53
‐UHDL with bolus	9.18	11.08	10.16	11.73

## 4 DISCUSSION

The mobile CBCT system (ImagingRing, Elekta) has been developed to provide in‐room volumetric images. With the in‐room imaging guidance, the efficiency and accuracy of brachytherapy can be further improved. Several studies have already demonstrated the potential use of this mobile CBCT for brachytherapy applicator placement verification and treatment planning.^22^, ^23^ Patients can stay in the same room for applicator placement and brachytherapy treatment without being transferred to another room for CT image acquisition.

This study evaluated the system using manufacturer‐defined standard imaging protocols with AEC for HDR gynecologic brachytherapy patients, without manual adjustment of scan parameters. Specifically, we comprehensively evaluate the HDR gynecologic imaging protocols using the CatPhan phantom, while other site‐specific protocols were not included. Previous studies have suggested that protocol‐specific parameter adjustment may improve image uniformity and artifact suppression depending on the scan object.^14^, ^24^ Depending on the clinical applications, future investigations would incorporate other site‐specific protocols in conjunction with AEC for the image quality evaluation.

This system incorporates AEC to dynamically adjust the x‐ray tube current based on the attenuation characteristics of the scanned object. The findings of this study demonstrated that AEC functioned effectively within the MDL protocol, modulating real‐time tube current according to phantom attenuation properties. Even when the initial mA preset was high, AEC dynamically adjusted the current to optimize imaging conditions. When the phantom was wrapped with a bolus to simulate increased tissue thickness, the real‐time current increased accordingly, highlighting the AEC system's ability to compensate for greater attenuation. The CBDI_w_ values also varied in response to changes in real‐time current, as thicker phantoms required higher radiation doses to maintain image quality.

The evaluation of NPS demonstrated that the UHDL protocol exhibited lower NPS values compared to the MDL protocol. This finding aligns with the image noise analysis, which showed that the UHDL protocol resulted in greater noise levels than the MDL protocol. The increased noise in the UHDL protocol contributed to its higher NPS values at low spatial frequencies.

Analysis of the MTF revealed that all tested conditions exhibited reduced MTF values at high spatial frequencies, indicating limitations in edge sharpness and spatial resolution. Overall, the MTF curves in both protocols remined similar with or without a bolus. This is important clinically to obtain similar spatial resolution for different BMIs.

The assessment of image sensitometry confirmed that HU linearity remained consistent across BMI categories in both the MDL and UHDL protocols. The *R* ^2^ values remained high, indicating a strong correlation between measured and expected HU values. Notably, the MDL protocol maintained HU linearity both with and without a bolus, despite variations in tube current modulation. This is an important observation for using the ImagingRing CBCT system for future investigations of using for model‐based dose calculation algorithms such as Advanced Collapsed cone Engine in Oncentra (Elekta) or Acuros in BracyVision (Varian) to calculate the absorbed dose to heterogeneous tissues.^25^, ^26^

**FIGURE 11:**
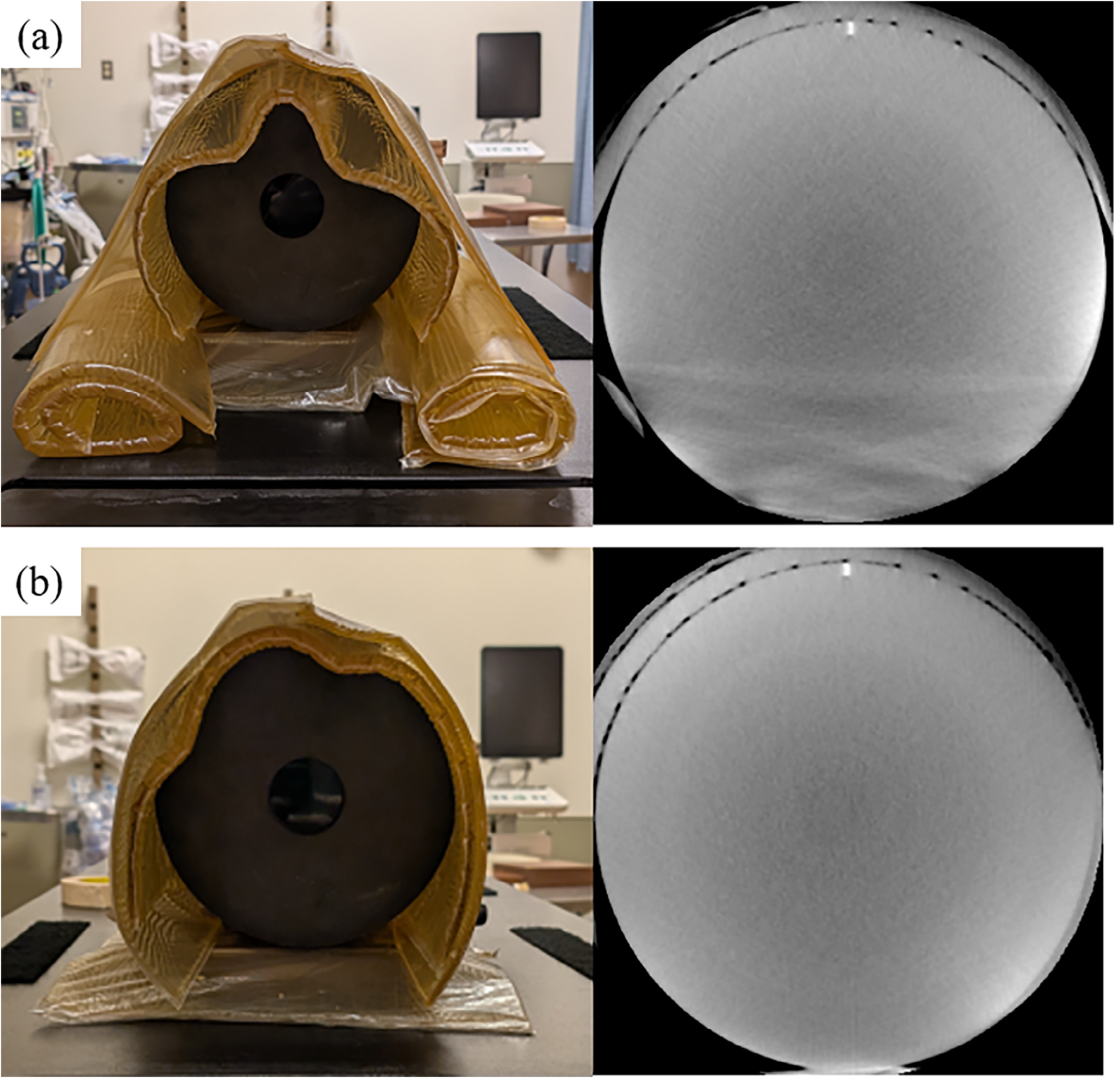
The experiment setup and the CT images in the uniformity module in the MDL protocol: (a) is a full bolus, (b) is a half bolus. (WL/WW [‐40, 500]).

However, the image quality is degraded when scanning large objects. As shown in Figure 9, there are streak artifacts on the bottom of the image of Catphan phantom wrapped with bolus. The streak artifacts were observed in the bottom area mainly due to photon starvation. Another scan was performed by removing the side bolus, and no significant streak artifacts were observed as shown in Figure 11. Image noise is also increasing on the Catphan images with bolus. Even UHDL protocol is used, the image quality remains limited, which might impact the clinical applications. Similar findings were reported in the previous studies. For example, Karius et al. performed the anthropomorphic phantom experiment; however, they could not obtain good image quality even if they used the protocol which obtained the best image quality with a Catphan phantom. The anthropomorphic has larger size than the Catphan phantom with a diameter of 20 cm.^14^ Enhancing image quality during the scanning of large objects is essential.

Furthermore, this study demonstrated the performance of AEC using the Catphan phantom, which does not include any metallic material. In recent years, MR imaging has become widely adopted for HDR brachytherapy due to its superior soft tissue contrast, and many contemporary MR/CT compatible applicators are designed without metallic components. However, certain applicators, such as steel needles or shielded applicators, still contain metal elements. These materials may introduce beam hardening and streak artifacts, which would potentially affect the image quality and clinical use. Previous studies have highlighted these issues.^14^ To address these issues, the metal artifact reduction (MAR) algorithm is provided by the vendor and is available when metallic components are within the scan range. Future studies would incorporate anthropomorphic phantoms with metallic inserts to further assess AEC response and image quality with MAR in clinically realistic scenarios.

Overall, these results highlight the effectiveness of AEC in optimizing radiation dose and image quality in a recently introduced mobile CBCT system for brachytherapy. The MDL protocol successfully adjusted tube current, maintaining image quality while reducing radiation dose. This makes it particularly suitable for applicator placement verification. In contrast, the UHDL protocol achieved the highest image quality, making it the preferred choice for brachytherapy treatment planning.

## 5 CONCLUSION

This study demonstrated that AEC effectively modulates tube current in real time, optimizing radiation dose and image quality based on x‐ray attenuation. The MDL protocol maintains image quality comparable to the UHDL protocol while reducing radiation exposure, particularly for thinner patients. However, image quality is slightly degraded when scanning large objects. The UHDL protocol delivers the highest image quality at the maximum available exposure of the machine, making it the recommended choice for brachytherapy treatment planning. These findings support the safe and effective use of mobile CBCT in brachytherapy, highlighting the advantages of AEC for imaging dose efficiency.

## AUTHOR CONTRIBUTIONS

Keita Okazaki wrote the first draft of the manuscript and analyzed image quality using Python. Yingxuan Chen, Keita Okazaki and Wenchao Cao performed the experiment using a CatPhan phantom and ImagingRing CBCT system. Other authors contributed to the critical review and revision of the manuscript, providing valuable feedback. Yingxuan Chen provided overall guidance and leadership of the study through discussions with the other authors, reviewed the results, and reviewed the manuscript.

## CONFLICT OF INTEREST STATEMENT

The authors declare no conflicts of interest
